# Three-Dimensional Manipulation of Micromodules Using Twin Optothermally Actuated Bubble Robots

**DOI:** 10.3390/mi15020230

**Published:** 2024-01-31

**Authors:** Liguo Dai, Lichao Liu, Yuting Zhou, Aofei Yan, Mengran Zhao, Shaobo Jin, Guoyong Ye, Caidong Wang

**Affiliations:** 1 Henan Provincial Key Laboratory of Intelligent Manufacturing of Mechanical Equipment, Zhengzhou University of Light Industry, Zhengzhou 450002, China; dailg@zzuli.edu.cn (L.D.); 332202040181@email.zzuli.edu.cn (L.L.); 332304040433@email.zzuli.edu.cn (A.Y.); 2021846@zzuli.edu.cn (M.Z.); 2021030@zzuli.edu.cn (S.J.); 2 State Key Laboratory of Robotics, Shenyang Institute of Automation, Chinese Academy of Sciences, Shenyang 110016, China; zhouyuting@sia.cn; 3 Institutes for Robotics and Intelligent Manufacturing, Chinese Academy of Sciences, Shenyang 110016, China; 4 University of Chinese Academy of Sciences, Beijing 100049, China

**Keywords:** bubble, robots, micro-manipulation, optothermal effect, beam splitting

## Abstract

A 3D manipulation technique based on two optothermally generated and actuated surface-bubble robots is proposed. A single laser beam can be divided into two parallel beams and used for the generation and motion control of twin bubbles. The movement and spacing control of the lasers and bubbles can be varied directly and rapidly. Both 2D and 3D operations of micromodules were carried out successfully using twin bubble robots. The cooperative manipulation of twin bubble robots is superior to that of a single robot in terms of stability, speed, and efficiency. The operational technique proposed in this study is expected to play an important role in tissue engineering, drug screening, and other fields.

## 1. Introduction

Microrobotics has developed rapidly in recent decades, owing to various driving and control technologies. For instance, chemical [1,2,3], magnetic [4,5,6], acoustic [7,8,9], electric [10,11,12], and optical energy [13,14,15] can be used to promote and control the movement of microrobots. Among these, lasers have the advantages of high density, high precision, and high wireless transmission efficiency [16,17,18]. In addition, microbubbles, which can be actuated optically, play an increasingly important role in microfluidics and microoperation [19,20,21]. Their properties, such as controllability, compressibility, and insolubility, make them widely applicable for operating microparticles, micromodules, and cells [22,23,24].

At the micro/nanoscale, several methods have been used to generate and control bubbles for microoperation, including injection [25,26], electrowetting [27,28], and optothermal effects [29,30]. Bubbles injected by microtubules are difficult to control, and the bubbles on electrowetting electrodes lack flexibility. Thus, optothermally actuated bubbles are most widely used in the manipulation of micro-objects. Generating bubbles involves concentrating a beam of light, usually a laser, on a point on a chip. This process causes the absorbing surface to absorb the laser energy and convert it into heat, thereby heating the targeted region and the surrounding fluid almost entirely [29,31]. When a bubble is formed, the convective flow caused by the temperature gradients and particles is attracted and adheres to the bubble surface. When the laser is moved, the microbubble follows the light and moves on the solid–liquid interface, and the trapped microspheres and cells can be transported by the bubble robot. In addition, larger objects such as micromodules in contact with bubbles can be pushed or pulled directly by the moving bubble robots. When bubbles are generated under micromodules, the microrobot can manipulate and assemble micro-objects in a three-dimensional (3D) space and not just in a two-dimensional (2D) plane [32]. Complex integrated assembly can also be carried out by utilizing the rapid growth and slow disappearance characteristics of bubble robots [33]. In tissue engineering, microrobots have been used to operate and assemble hydrogel modules containing cells to obtain biomimetic tissues in vitro [34]. However, the manipulation ability and efficiency of bubbles driven by a single laser beam are relatively low. One improvement measure is to produce and drive multiple microrobots simultaneously. To focus the laser at different locations on the absorbing layer of the substrate, a dual-axis scanning mirror system (DASMS) or programmable spatial light modulator (SLM) can be integrated into the optical system [35,36]. Multiple separated beams can produce multiple hot spots on the liquid–solid interface, which, in turn, can generate and drive multiple bubbles. Although lasers can be divided into multiple beams using DASMS [35] or SLM [36], these methods require software and prior parameter-setting, which is inconvenient.

In this study, a novel and more direct laser beam-splitting method is proposed for the actuation of multiple bubble robots. A beam-splitting device consisting of two splitters and mirrors was utilized to obtain two laser beams from a single laser source, and to vary the spacing of the beams. The proposed beam-splitting method is more intuitive and direct than either DASMS or SLM, and requires neither complex operations nor prior settings. Twin microbubbles can be generated and actuated simultaneously using the optical system. In addition, these synchronous bubble robots can manipulate resin micromodules in both the 2D plane and 3D space. Compared to technology reliant on a single bubble robot, the manipulation achieved using twin bubble robots is more stable, rapid, and efficient.

## 2. Materials and Methods

### 2.1. Beam-Splitting Device and Method

**Figure 1:**
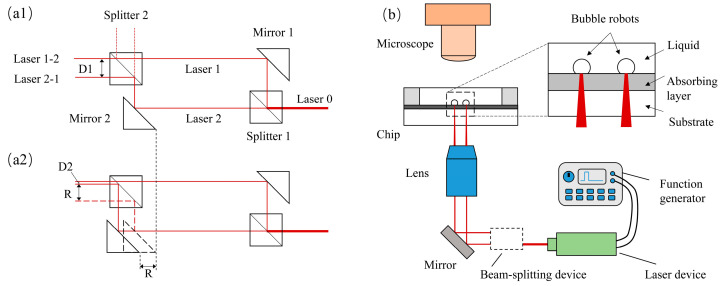
Schematic of the experiment system. (**a1**) The beam-splitting device consisted of two splitter cubes and prism mirrors before spacing adjustment. (**a2**) Schematic diagram after spacing adjustment. (**b**) The experimental setup.

In this study, two non-polarizing beam splitters and a pair of mirrors were used to construct a beam-splitting device. This device can split a laser beam into two parallel beams and easily change the distance between them (Figure 1a).

Non-polarizing beam-splitter cubes (BS014, Throlabs Inc., Newton, NJ, USA) were fabricated from N-BK7. A dielectric beam splitter coating was applied to the hypotenuse of one of the two prisms that comprise the cube. The splitters were designed so that part of the light was reflected at a 45° angle, while the remaining light was transmitted (Figure 1(a1)). The splitting ratio (reflectance: transmission) provided by the splitter in this study was 50:50; in other words, the amounts of reflected and transmitted light were almost the same. The undivided laser beam was denoted as Laser 0. The reflected beam (Laser 1) and transmitted beam (Laser 2) were redirected by right-angle prism mirrors (MRA25-E03, Throlabs Inc., Newton, NJ, USA) with broadband dielectric coatings, and subsequently entered the second non-polarizing splitter at different angles (90° between them). This resulted in the emission of two pairs of parallel laser beams in two directions. Laser 1-2 (the transmitted beam of Laser 1) and Laser 2-1 (the reflected beam of laser 2) were employed to produce and drive the bubble robots. Another pair of laser beams (the reflected beam of Laser 1 and the transmitted beam of Laser 2, represented by dotted lines in the diagram), which are not labeled in the image, were abandoned. The splitter cubes and prism mirrors were cube-mounted in cage cubes (CCM1-4ER/M, Throlabs Inc., Newton, NJ, USA) to form a cage system, i.e., a beam-splitting device.

To generate and drive bubble robots more easily and conveniently, the distance between the two laser beams emitted by the beam-splitting device must be controllable. By moving the mirror at position 2 (Mirror 2), the relative distance between the two beams of light converging on the second beam splitter (Splitter 2) can be varied (Figure 1(a2)). When Mirror 2 was moved a distance (R) using manual linear staging, the position of Laser 2 after being reflected changed. Consequently, the position of Laser 2-1 passing through Splitter 2 also changed. The distance between the two split beams changed from D1 to D2. The change in the beam distance is equal to the distance the mirror moved, that is, the difference between D1 and D2 is R. In reality, a change in the position of either optical element (the beam splitter or mirror) can change the beam distance. Consequently, both the positions and distances of the two laser beams could be controlled. The lasers could be moved synchronously by moving the optical equipment (laser, beam splitter, mirror, and lens), and the beam-splitting device could be used to control the distance between the two laser beams. The experimental setup is shown in Figure S1.

### 2.2. Experimental Setup

The devices used in this study are shown in Figure 1. As illustrated in Figure 1b, the chip consists of a substrate and an absorbing layer. Metallic titanium (Ti) film was sputtered onto a glass sheet using a magnetron sputtering instrument. The thickness of the titanium film for light absorption was 50 nm, and the substrate was silica glass 1.2 mm in thickness. The wavelength of the laser used to produce and drive the bubbles was 980 nm. After passing through the beam-splitting device, the laser beam was split into two beams, which were shone onto the chip to actuate the bubble robots in parallel. To increase the energy density of the laser, a lens (25×, NA = 0.40) was used to focus the split laser beams onto the absorbing layer. A mirror was placed between the beam-splitting device and the lens so that the two laser beams could be installed horizontally, effectively reducing the total height of the experimental system. The broadband dielectric mirror (BB1-E03, Throlabs Inc., Newton, NJ, USA) had a high reflectivity of near-infrared (NIR) light and could be matched to the parameters of the laser. To move the two laser beams, the laser device, beam-splitting device, mirror, and lens were connected to a 3-axis stage (not shown in the schematic). In addition, pulse-width modulation (PWM) waves produced by a waveform generator (ArbStudio 1102, Teledyne LeCroy Inc., Chestnut Ridge, NY, USA) were used for intermittent laser irradiation. A microscope connected to a computer was used to observe the laser spots, bubble robots, and manipulated micro-objects.

### 2.3. Fabrication of Micromodules

The micromodules were fabricated by a micro–nano 3D printing system. First, the shape and size of the micromodules were designed using SOLIDWORKS software (Educational version). A mesh was generated and exported as an STL file for printing. The STL file was then imported into the DeScribe software (version number: 2.5.7, Nanoscribe GmbH, Karlsruhe, Germany), which enabled us to display a 3D preview of the model and set writing parameters, including rescaling, structure slicing, and plane filling lines, which represent the laser scanning paths. The micromodules were 3D microprinted using a two-photon laser direct writing system (Photonic Professional GT2, Nanoscribe GmbH, Germany) with an objective (25×, NA = 0.8) and IP-S resin, a material produced by Nanoscribe, on a glass substrate. The 2-PP printing was performed in oil mode. A femtosecond laser with a wavelength of 780 nm was used as the light source. Micromodules were developed using propylene glycol methyl ether acetate solution (PGMEA) for 20 min and isopropanol (IPA) for 30 s to remove the remaining resin solution. The micromodules were scratched off the substrate using a pipette tip and transferred to the chip.

## 3. Results

In the experimental system, including the beam-splitting device, two laser beams shone on the chip and could be employed for the generation of bubbles. When the two laser beams moved together, the twin bubbles could be driven and moved simultaneously on the surface of the light-absorbing layer. The 2D operation of the micromodule could be accomplished using two mobile bubble microrobots. The directions of movement of the bubble robots and micromodules were variable. In addition, 3D rotation of micro-objects could be achieved when bubble robots were generated and moved under the micromodule. The bubble robots kept the rotated micromodules stable and mobile.

### 3.1. Generation and Driving of Two Microbubbles

#### 3.1.1. Detection and Analysis of Laser Beams

To use two lasers to drive two bubbles in parallel, the intensity and size of the two lasers should be as similar as possible. After splitting, the two laser beams were measured using a beam-profiling camera (SP928, Ophir Optronics Solutions Ltd., Har Hotzvim, Jerusalem, Israel). Optical measuring instruments can capture and analyze light accurately and are capable of measuring the profiles of continuous and pulsed lasers.

**Figure 2:**
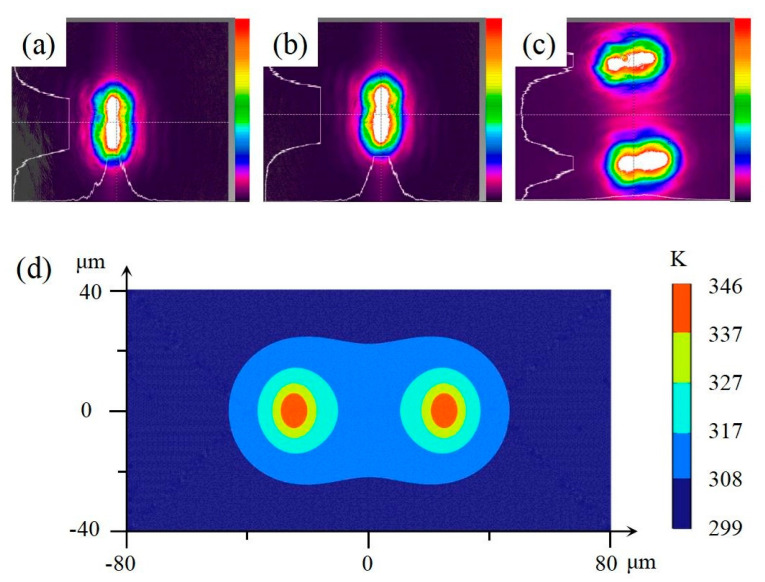
Profiles of the laser beams. (**a**) Two-dimensional profile of Laser 1−2. (**b**) Two-dimensional profile of Laser 2−1. (**c**) Two-dimensional profiles of the two laser beams. (**d**) Simulation results of optother-mal effects caused by two beams.

The intensities and profiles of the two laser beams are listed in Table 1 and shown in Figure 2. The 2D profiles of Laser 1-2 and Laser 2-1 are shown in Figure 2a,b, respectively. When one laser beam was measured, the other laser beam was blocked. The 2D of the two laser beams without occlusion are shown in Figure 2c. The laser beam intensities are listed in Table 1. Although the profiles of the two laser beams were found to be very similar when the beam-splitting device was used, the intensities were slightly different. The light-intensity ratio was approximately 1.13:1. The light intensity values were relatively stable; that is, the standard deviations (SD) of light intensity were relatively small. After splitting, the two laser beams do not affect each other and can be measured and observed simultaneously, as shown in Figure 2c. When two laser beams were present simultaneously, the intensity was equal to the sum of the two beams, and the SD was smaller than that of either beam. Irrespective of the number of laser beams (single beam or double) the peak intensities were very similar and depended on the parameters of the laser device. The 2D profiles of the laser beams were both elliptical rather than standard circles, primarily because of the effect of the splitters.

After the absorbing layer converted the light into heat energy, the substrate was heated via conduction. COMSOL Multiphysics software was used to simulate the temperature distribution at the interface of the film and when the laser beams were illuminated (Figure 2d). Each laser beam generates a heat source, and the two heat sources are relatively independent. The temperature was highest at the two laser focal points and then gradually decreased in all directions. In the middle of the focus area, the temperature was slightly higher than that in the other areas. When the distance between the two laser beams was sufficient, two independent thermal fields could be generated; therefore, two bubbles could be produced independently.

#### 3.1.2. Generation and Driving of Two Microbubbles

As with a single laser beam, when two laser beams hit the surface of an absorbing layer, part of the light is reflected or passes through the absorbing layer, and part of the light is absorbed. The absorptivity and transmissivity in the experiment were affected by the parameters of the chip and laser, such as the laser wavelength and the thickness and material of the coating film. The absorbed energy was converted into heat, which was used to heat chips and fluids. As the temperature increased, the gas dissolved in the water precipitated out and formed air bubbles.

**Figure 3:**
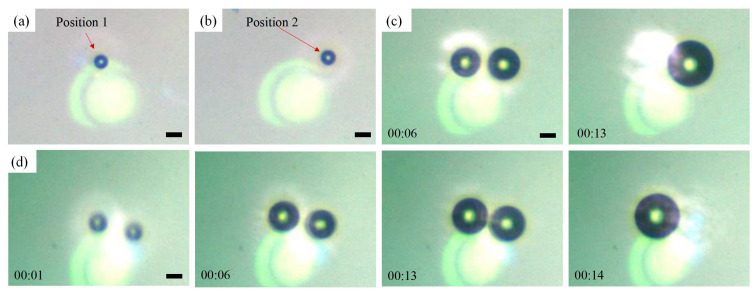
Generation of optothermal bubbles. (**a**,**b**) Single bubble generated in Position 1 and Position 2. (**c**) Growth and merging of two bubbles. (**d**) Generation, growth and merging of two bubbles. Scale bars: 20 μm.

When two parallel laser beams are focused on the chip simultaneously, the generation of two bubbles simultaneously largely depends on the beam spacing. If the distance is sufficiently large, the two independent thermal fields do not affect each other, but produce two bubbles in parallel. Conversely, if the spacing is too small, the two lasers will work together, resulting in the creation of a larger bubble. That is, two small bubbles may merge during the initial stage of formation if the spacing is insufficiently large. Bubbles may also merge as they grow, even if the beam spacing is large. The two bubbles in the experiment were generated separately, and their growth and merging are shown in Figure 3.

In Figure 3a,b, two laser beams are illuminated on the chip, with one at a time being masked to produce two separate bubbles one after another. During this time, the optics remained stationary; therefore, the two laser beams were focused at different locations, corresponding to the positions of the two resulting bubbles. When the two lasers worked simultaneously, two bubbles were produced simultaneously (Figure 3c). As the laser continued to irradiate, the sizes of the two bubbles increased until eventually they coalesced into a single large bubble. The merged bubble can continue to grow as long as the lasers continue to shine. During merging, the bubbles in both positions can be swallowed, as shown in Figure 3c,d. Both bubbles have the chance to persist: that is, the new bubble occupies one of the original two positions of the bubbles, rather than appearing in the middle of the laser spots. The partially visible circular spot in the image represents the surface of the focusing lens. The time shown in the figure was measured from the moment the bubble was created. The frequency and duration of laser irradiation can be controlled using PWM waves [17]. The lasers in Figure 3 used a duty cycle of 20%. When the duty cycle was small, for example, 10%, the growth rate of the bubbles could be significantly reduced, and the two bubbles could be maintained at an appropriate size for subsequent micro-operation.

**Figure 4:**
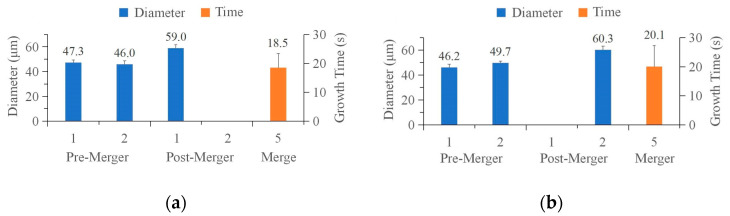
The size of the bubble before and after merging, and the time of merging. The bubbles at (**a**) Position 2 and (**b**) Position 1 disappear.

Typically, a smaller bubble is swallowed by a larger one, as shown in Figure 4. In Figure 4a, the bubble at Position 1, the focal point of Laser 1-2 (shown in Figure 3a), is larger, and the merged bubble is still in the position. Conversely, when the bubbles at Position 2 (Figure 3b) are larger, the bubbles at Position 1 disappear after merging, as shown in Figure 4b. The first two columns in Figure 4 show the bubble sizes before the merger, the third and fourth columns show the bubble sizes after the merger, and the last column shows the bubble growth time during the merger. At the time of merger, both bubbles were approximately 45–50 μm in diameter. The distance between the focal points of the two beams was about 50 μm, indicating that the two bubbles with similar size would merge just before they came into contact. Furthermore, the volume of the merged bubbles was approximately equal to the sum of the volumes of the first two bubbles. The growth time for both bubbles was approximately 20 s, and the diameter of the merged bubbles was approximately 60 μm. The SD of the time from generation to merging was relatively large because of the fluctuations in the growth rate of the bubbles.

**Figure 5:**
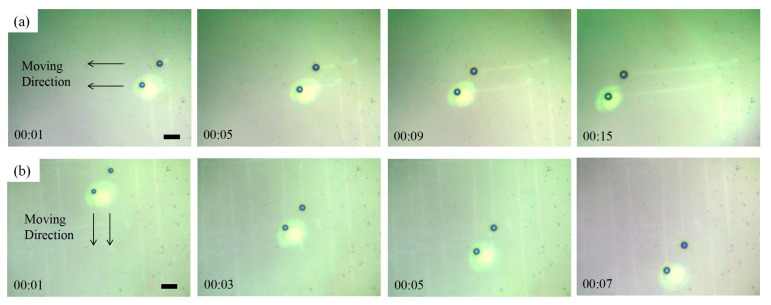
(**a**) Synchronous motion to the lift of twin bubbles. (**b**) Synchronous downward motion of twin bubbles. Scale bars: 50 μm.

As with a single bubble driven by a single laser beam, two bubbles can be driven by two laser beams. As shown in Figure 5, the two bubbles in the experiment move synchronously in different directions. In Figure 5a,b, the movement directions are perpendicular to each other. If the laser beams move in other directions, the bubbles follow them. The movement distances of the bubbles in Figure 5a,b are 320 and 310 μm, with corresponding velocities of 21.3 and 44.3 μm/s, respectively. The speed of the bubbles depends primarily on the laser beam. In the process of moving, the bubbles may fail to keep up with the laser, remain in place, or float up in the water, after which a new bubble appears. When heated by the focused lasers, some traces were left on the Ti film, as shown in the images.

### 3.2. Manipulation by Bubble Robots

#### 3.2.1. Two-Dimensional Manipulation

A single bubble microrobot can facilitate 2D operation of the micromodules. Because there is only one contact point between the robot and micro-object, the micromodule may deflect significantly during movement; thus, the stability and efficiency are relatively low. When the number of bubble robots is increased to two, the stability of the micromanipulation improves.

**Figure 6:**
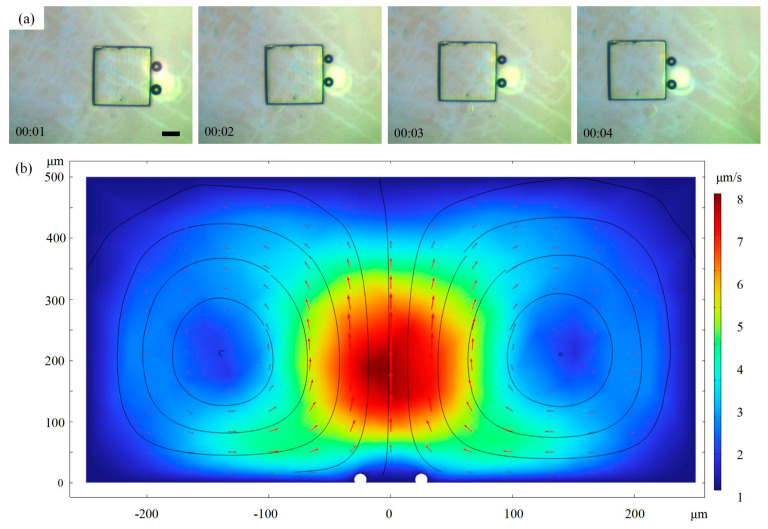
(**a**) Two-dimensional motion of micromodule actuated by twin bubbles. Scale bars: 50 μm. (**b**) Simulation results of flow around two bubbles.

Micromodules can move using two bubble microrobots, as shown in Figure 6a and Movie S1. Two parallel bubble robots were used to push the micromodules back and forth by changing their corresponding positions to change the motor direction of the micromodule. Consequently, the operation remained slow when the direction of motion needed to be switched. The movement of the micromodule was approximately 96 μm, and the velocity was 24 μm/s. At least four bubble microrobots were required to achieve fast operation in all directions. Overall, the operation method based on two bubble robots was superior to that based on a single robot in terms of stability and efficiency, but it requires further development.

Although two bubbles were produced under the guidance of two lasers, the flow around them was similar to that around a single bubble [32,37]. The circulation was axisymmetrically distributed in the middle of the twin bubbles (Figure 6b). In the lower fluid region, the fluid flowed towards the middle of the bubble, the flow velocity was low, and the bubbles had no obvious effect on the velocity and direction of the flow. In the upper region between the two bubbles, the fluid flowed upwards at the highest velocity. In the middle region, the fluid flowed upwards, which proved that the flow field could indeed provide the micromodule with an upward force and make the micromodule complete a three-dimensional flip. The entire fluid region exhibited circular flow.

#### 3.2.2. Three-Dimensional Manipulation

In tissue engineering, micromodules that encapsulate cells are manufactured, manipulated, and assembled into 3D structures similar in shape to biological tissue. The 3D operations are expected to exhibit high efficiency and wide applicability. Two bubbles can produce a larger flow field than a single bubble, which makes it easier to operate a larger module.

**Figure 7:**
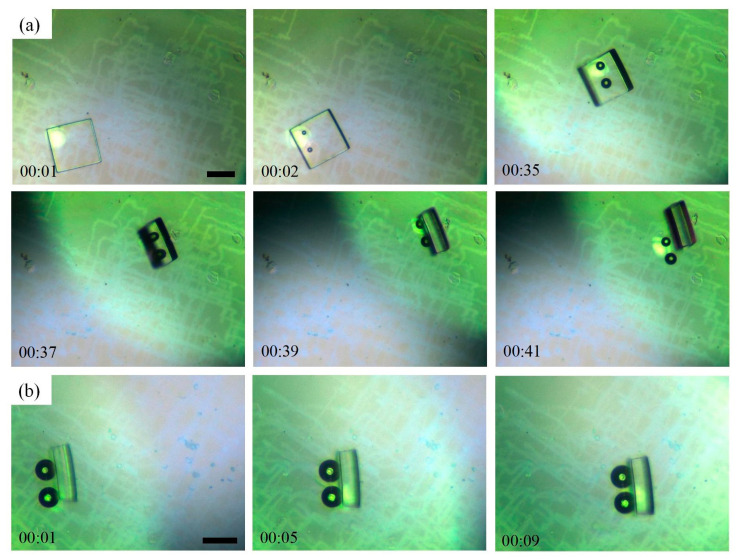
(**a**) Three-dimensional manipulation of a micromodule using two bubble robots under-neath it. (**b**) Two-dimensional movement of the micromodule that had been manipulated in 3D previously. Scale bars: 100 μm.

Two bubble robots are used to complete the 3D operation of the micromodule, as shown in Figure 7 and Movie S2. The resin micromodules were 200 μm in length and width, and 50 μm in height. In 2D operation, bubble robots were generated and moved around the micromodule. In 3D operation, the focal points of the lasers were directed below the micromodule, creating bubbles underneath it. The bubbles and the surrounding flow fields effectively lifted the resin module. As the bubbles moved, the flow fields changed, and the micro-object was completely lifted. The 3D operation of the micromodule, which is much larger than those of the bubble robots, can be obtained from Figure 7a. The flipped micromodule could stand stably and make contact with the chip using its small side area. The standing micromodule can also be moved in a 2D plane (Figure 7b). The size of the micro-objects that can be operated on using two microrobots is significantly better than that of systems using one microrobot, which expands the potential applications of bubble robots. Multimodules can be assembled into 3D structures with specific shapes. Owing to the large amount of movement of the lasers, more traces were left on the chip; however, this did not affect their continued use.

## 4. Conclusions

In this study, a manipulation technology using twin optothermally actuated bubble robots was proposed. To produce two bubbles simultaneously, a laser beam must undergo splitting, reflection, and splitting to obtain two pairs of parallel laser beams. Although only one group of lasers can be used, this method allows for faster and more direct control of the position and spacing of the lasers. The high power intensities promoted the generation of bubbles when the two laser beams were focused on the absorbing layer of the chip. An appropriate spacing between the laser beams was selected. In addition, PWM waves were introduced to control the growth rate of the bubbles. Optothermally generated bubbles were used to operate the micromodules. The results show that using two bubble robots simultaneously is superior to using single robots in terms of efficiency, applicability, and stability. This manipulation technology is expected to play an important role in a number of fields including biology and medicine.

## Figures and Tables

**Table 1:** Intensities of split laser beams.

	Laser 1-2	Laser 2-1	Laser 1-2 and 2-1
Mean ^1^	5.86 × 10^8^	6.64 × 10^8^	1.22 × 10^9^
SD ^1^	1.56 × 10^5^	2.16 × 10^5^	6.97 × 10^4^

^1^ Unit of data: counts.

## Data Availability

Data available on request from the authors.

## Supplementary Materials

The following supporting information can be downloaded at: https://www.mdpi.com/article/10.3390/mi15020230/s1, Figure S1. Actual experiment setup. (a) The whole optical part including laser, objective lens and beam-splitting device. (b) Beam-splitting device; Movie S1: 2D motion of micromodule actuated by twin bubbles; Movie S2: 3D operation of micromodule actuated by twin bubbles.
