# Supplementary material for: Three-Dimensional Manipulation of Micromodules Using Twin Optothermally Actuated Bubble Robots
Source: Micromachines (Basel). 2024 Jan 31;15(2):230. doi: 10.3390/mi15020230 (PMC10892707; doi:10.3390/mi15020230)
Supplement: Supplementary file 1 [file micromachines-15-00230-s001.zip › micromachines-2780345-supplementary.pdf]

Supplementary

Three-Dimensional Manipulation of Micromodules Using Twin Optothermally Actuated Bubble Robots

Liguo Dai [1], Lichao Liu [1], Yutting Zhou [2,3,4], Aofei Yan [1], Mengran Zhao [1], Shaobo Jin [1], Guoyong Ye [1,*] and Caidong Wang [1,*]

[1] Henan Provincial Key Laboratory of Intelligent Manufacturing of Mechanical Equipment, Zhengzhou University of Light Industry, Zhengzhou 450002, China; dailg@zzuli.edu.cn (L.D.); 332202040181@email.zzuli.edu.cn (L.L.); 332304040433@email.zzuli.edu.cn (A.Y.); 2021846@zzuli.edu.cn (M.Z.); 2021030@zzuli.edu.cn (S.J.)
[2] State Key Laboratory of Robotics, Shenyang Institute of Automation, Chinese Academy of Sciences, Shenyang 110016, China; zhouyuting@sia.cn
[3] Institutes for Robotics and Intelligent Manufacturing, Chinese Academy of Sciences, Shenyang 110016, China
[4] University of Chinese Academy of Sciences, Beijing 100049, China
* Correspondence: 2021071@zzuli.edu.cn (G.Y.); 2011009@zzuli.edu.cn (C.W.)

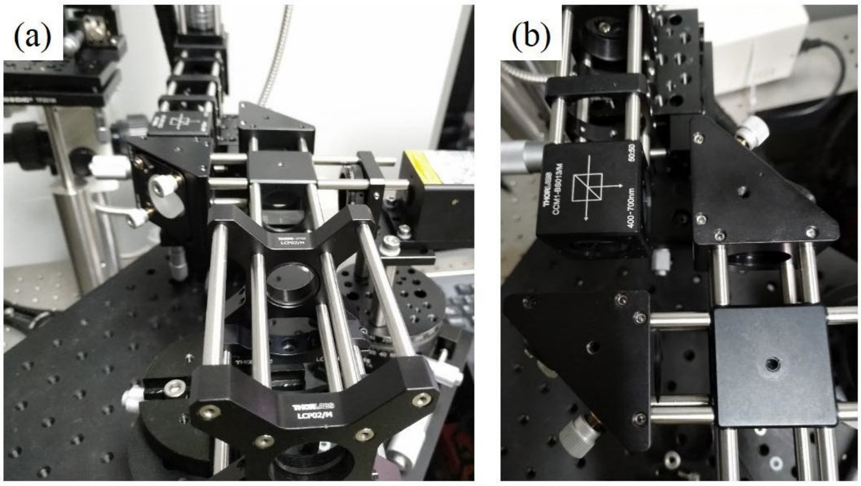

Figure S1. Actual experiment setup. (**a**) The whole optical part including laser, objective lens and beam-splitting device. (**b**) Beam-splitting device.
